# Supplementary material for: Cuban Sugar Cane Wax Alcohol Exhibited Enhanced Antioxidant, Anti-Glycation and Anti-Inflammatory Activity in Reconstituted High-Density Lipoprotein (rHDL) with Improved Structural and Functional Correlations: Comparison of Various Policosanols
Source: Int J Mol Sci. 2023 Feb 6;24(4):3186. doi: 10.3390/ijms24043186 (PMC9965296; doi:10.3390/ijms24043186)
Supplement: Supplementary file 1 [file ijms-24-03186-s001.zip › ijms-2162130-supplementary.pdf]

출력일자 : 2020년 02월 20일

수입식품등 검사결과

정보공개용 자료

수입 신고인 (수입 화주)	상 호	(주)레인보우앤네이처코리아	대표자	이병구
	주 소	서울특별시 서초구 강남대로39길 15-10 , 2층 205호(서초동, 한라비발디스튜디오)		

접수번호	202000055987	접수일자	2020년 02월 10일
제 품 명	POLICOSANOL SUGAR CANE WAX ALCOHOL	제품한글명	폴리코사놀사탕수수왁스알코올
검사종류	무작위표본	제품유형	개별인정형건강기능식품
의뢰기관	서울청 수입관리과		
의뢰번호	202000055987		
검사기관	한국식품산업협회 부설 한국식품과학연구원		
판 정	적합		

적용 기준 및 규격

시험ㆍ검사 항목명	규격	결과	판정	비고
총 지방족 알코올	900.0 이상	982.9	적합	
1-테트라코사놀	0.1 이상 20.0 이하	0.3	적합	
1-헥사코사놀	30.0 이상 100.0 이하	38.7	적합	
1-heptacosanol	1.0 이상 30.0 이하	8.6	적합	
1-nonacosanol	1.0 이상 20.0 이하	5.9	적합	
1-triacontanol	100.0 이상 150.0 이하	139.4	적합	
납	1.0 이하	0.2	적합	
총 비소	1.0 이하	0.0	적합	
카드뮴	0.5 이하	0.0	적합	
총 수은	0.5 이하	0.0	적합	제조일자 2019.4.10 /유통기한 2024.4.1
대장균군	음성	음성	적합	
아세톤	0.03 이하	0.02	적합	
헥산	0.005 이하	0.001	적합	
1-dotriacontanol	50.0 이상 100.0 이하	78.4	적합	
옥타코사놀	600.0 이상 700.0 이하	691.7	적합	
지방알코올	1.0 이상 50.0 이하	19.8	적합	
성상	흰색(황색이 도는 흰색)의 미정질분말	적합	적합	

이 검사결과는 검사 의뢰된 검체에 한한 것으로, 검사결과를 허위ㆍ과대 표시ㆍ광고나 비방의 목적으로 사용하여서는 아니되며, 표시ㆍ광고에 사용하고자 할 경우에는 검사결과 전체 내용을 사실대로 표시ㆍ광고하여야 합니다.

2020년 02월 19일

한국식품산업협회 부설 한국식품과학연구원장

	sugarcane, 60%,Xian natural	sugarcane, 70%,Xian realin	ricebran, 98%,Shanxi
**Sample ID**	**11-22481**	**11-22483**	**11-22487**
Manufacturer	Xian Natural	Xian Realin	Shanxi
Source	Surga cane	Surga cane	Rice Brran
Total wax alcohol(mg/g) in the labe	600	700	980
1-tetracosanol(mg/g)	7.37	56.76	0.11
1-hexacosanol(mg/g)	89.12	95.17	5.51
1-heptacosanol(mg/g)	9.56	1.72	5.81
1-octacosanol(mg/g)	356	69.22	492.12
1-nonacosanol(mg/g)	12.06	8.74	1.83
1-triacontanol(mg/g)	132.73	236.99	12.63
1-dotriacontanol(mg/g)	3.08	74.26	not detected
1-tetratriacontanol(mg/g)	0.1	49.93	not detected
**Total wax alcohol(mg/g)**	**610.03**	**592.79**	**518.02**
Lead(mg/g)	not detected	not detected	not detected
Total Arsenic(mg/g)	not detected	not detected	not detected
cadmium(mg/g)	not detected	not detected	not detected
Total Mercury(mg/g)	not detected	not detected	not detected
Acetone(g/kg)	not detected	not detected	not detected
Hexane(g/kg)	0.0029	0.0049	0.0009
Coliforms	negative	negative	negative

한국식품과학연구원

Korea Advanced Food Research Institute<br>50, Botdeul-ro, Uiwang-si, Gyeonggi-do, Republic of Korea<br>TEL : 82-2-3470-8200 FAX : 82-2-523-2072 http://www.kafri.or.kr	KAFRI

Certificate of Laboratory Testing(Reference)

Receipt No.	2021-11-022481	Date of Receipt	2021.11.26
Product Name	Policosanol-Sugar cane wax alcohol 60 XN SAMPLE		
Client Company Name	RAINBOW AND NATURE KOREA CO., LTD		
Client Address	205, Hanravivaldi Bldg, 15-10, Gangnam-daero 39-gil, Seocho-gu, Seoul, Korea		
Client Name	LEE BYONG KU	Client Tel / Fax	02-3473-2371
Lot No.		Date of Manufacture /Expiration Date	/
Test Purpose	For confirmation(in company)	Date of Issue	2021.12.22

Test Items and Results

1-Hexacosanol(mg/g)................................89.12
1-Heptacosanol(mg/g)...............................9.56
1-Octacosanol(mg/g)................................356.00
1-Tetracosanol(mg/g)...............................7.37
1-Nonacosanol(mg/g)................................12.06
1-Triacontanol(mg/g)...............................132.73
1-dotriacontanol(mg/g).............................3.08
1-Tetratriacontanol(mg/g)..........................0.10
Total Wax Alcohols(mg/g)...........................610.03
Lead(mg/kg)..Not Detected
Total Arsenic(mg/kg)...............................Not Detected
Cadmium(mg/kg).....................................Not Detected
Total Mercury(mg/kg)...............................Not Detected
Acetone(mg/kg).....................................Not Detected
Hexane(mg/kg)......................................0.0029
Coliforms..Negative

* The above test items and results complied with the test method notified by MFDS.
* Policosanol test items and results complied with the test method notified by Certificate of Functional Ingredient For Health/Functional Food No.2006-4

Note: 1. The above merchandise was submitted and identified by the client.
2. The results shown in this report refer only to sample tested and it does not cover the quality of all products.
3. No one can use this report for the purpose of test, advertisement and litigation without KAFRI's consent.
4. This report has no legal effect.
5. This report is not related to KS Q ISO/IEC 17025 and KOLAS Accreditation.

This certificate does not comply with the Act on Testing and inspection of Food and Drugs of Ministry of Food and Drug Safe.

Lee kyoung bok

Testing Personnel

Testing Manager

Korea Advanced Food Research Institute

Korea Advanced Food Research Institute

Data File: C:\Projects\Functional Food\Result\policosanol\2021\1208.rslt\012.dat
Method: C:\Projects\Functional Food\Method\Policosanol-1.met
Acquired: 12/8/2021 9:33:11 PM (GMT +09:00)
Sample Id: 11-22481
Operator: kafri169

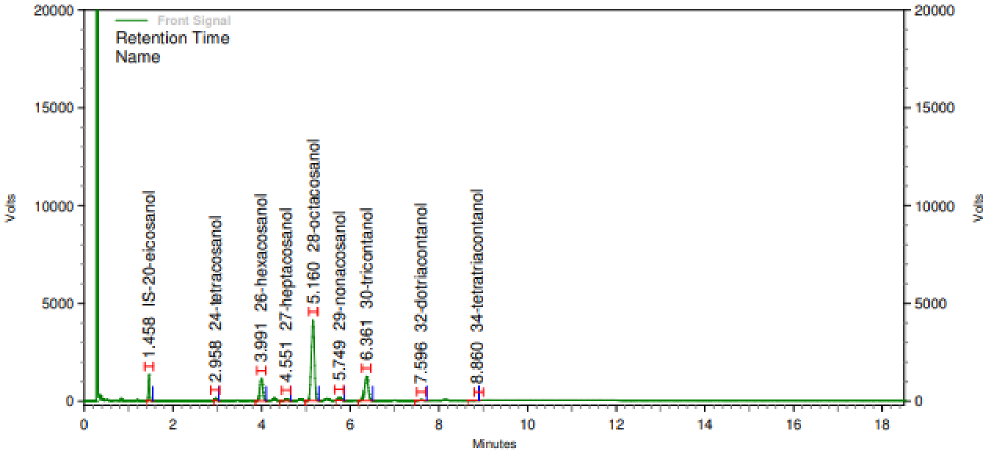

Front Signal Results

Name	Retention Time	Area
**IS-20-eicosanol**	1.458	21035640
**24-tetracosanol**	2.958	3245008
**26-hexacosanol**	3.991	38446905
**27-heptacosanol**	4.551	4336396
**28-octacosanol**	5.160	162442308
**29-nonacosanol**	5.749	5502512
**30-tricontanol**	6.361	58348677
**32-dotriacontanol**	7.596	1354444
**34-tetratriacontanol**	8.860	46077
**Totals**		294757967

한국식품과학연구원
Korea Advanced Food Research Institute

Korea Advanced Food Research Institute<br>50, Botdeul-ro, Uiwang-si, Gyeonggi-do, Republic of Korea<br>TEL : 82-2-3470-8200 FAX : 82-2-523-2072 http://www.kafri.or.kr	KAFRI

Certificate of Laboratory Testing(Reference)

Receipt No.	2021-11-022483	Date of Receipt	2021.11.26
Product Name	Policosanol-Sugar cane wax alcohol 70 XR SAMPLE		
Client Company Name	RAINBOW AND NATURE KOREA CO., LTD		
Client Address	205, Hanravivaldi Bldg, 15-10, Gangnam-daero 39-gil, Seocho-gu, Seoul, Korea		
Client Name	LEE BYONG KU	Client Tel / Fax	02-3473-2371
Lot No.		Date of Manufacture /Expiration Date	/
Test Purpose	For confirmation(in company)	Date of Issue	2021.12.22

Test Items and Results

1-Hexacosanol(mg/g)..............................95.17
1-Heptacosanol(mg/g).............................1.72
1-Octacosanol(mg/g)..............................69.22
1-Tetracosanol(mg/g).............................56.76
1-Nonacosanol(mg/g)..............................8.74
1-Triacontanol(mg/g).............................236.99
1-dotriacontanol(mg/g)...........................74.26
1-Tetratriacontanol(mg/g)........................49.93
Total Wax Alcohols(mg/g).........................592.79
Lead(mg/kg)......................................Not Detected
Total Arsenic(mg/kg).............................Not Detected
Cadmium(mg/kg)...................................Not Detected
Total Mercury(mg/kg).............................Not Detected
Acetone(mg/kg)...................................Not Detected
Hexane(mg/kg)....................................0.0049
Coliforms..Negative

* The above test items and results complied with the test method notified by MFDS.
* Policosanol test items and results complied with the test method notified by Certificate of Functional Ingredient For Health/Functional Food No.2006-4

Note: 1. The above merchandise was submitted and identified by the client.
2. The results shown in this report refer only to sample tested and it does not cover the quality of all products.
3. No one can use this report for the purpose of test, advertisement and litigation without KAFRI's consent.
4. This report has no legal effect.
5. This report is not related to KS Q ISO/IEC 17025 and KOLAS Accreditation.

This certificate does not comply with the Act on Testing and inspection of Food and Drugs of Ministry of Food and Drug Safe.

Lee kyoung bok
Testing Personnel

Testing Manager

Korea Advanced Food Research Institute

Korea Advanced Food Research Institute

Data File: C:\Projects\Functional Food\Result\policosanol\2021\1208.rslt\016.dat
Method: C:\Projects\Functional Food\Method\Policosanol-1.met
Acquired: 12/8/2021 10:58:34 PM (GMT +09:00)
Sample Id: 11-22483
Operator: kafri169

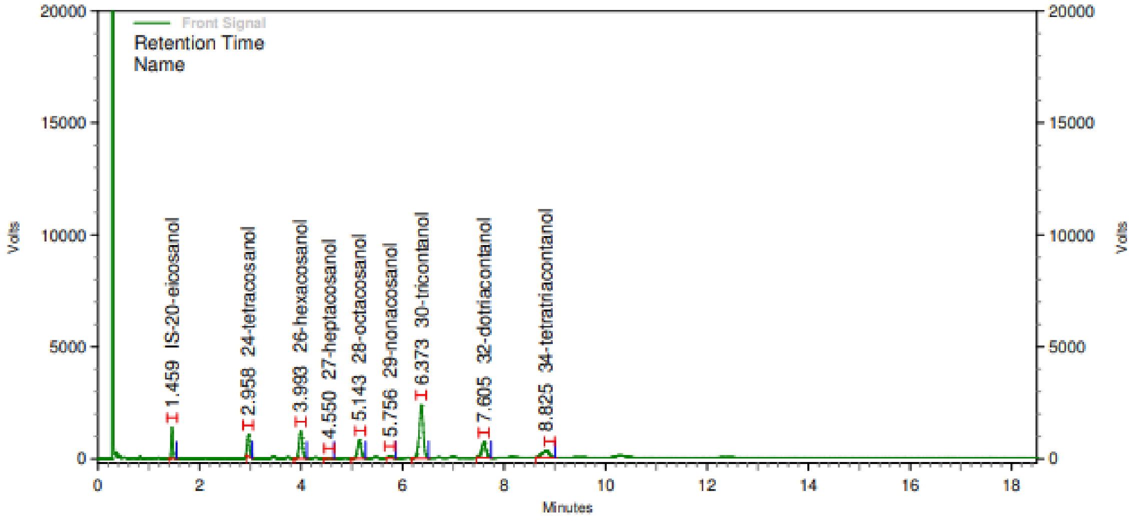

Front Signal Results

Name	Retention Time	Area
**IS-20-eicosanol**	1.459	21867381
**24-tetracosanol**	2.958	25061244
**26-hexacosanol**	3.993	41166689
**27-heptacosanol**	4.550	782670
**28-octacosanol**	5.143	31668026
**29-nonacosanol**	5.756	3997435
**30-tricontanol**	6.373	104458491
**32-dotriacontanol**	7.605	32732900
**34-tetratriacontanol**	8.825	22008969
**Totals**		283743805

한국식품과학연구원

Korea Advanced Food Research Institute<br>50, Botdeul-ro, Uiwang-si, Gyeonggi-do, Republic of Korea<br>TEL : 82-2-3470-8200 FAX : 82-2-523-2072 http://www.kafri.or.kr			KAFRI

Certificate of Laboratory Testing(Reference)

Receipt No.	2021-11-022487	Date of Receipt	2021.11.26
Product Name	Policosanol-Sugar cane wax alcohol RB 98S SAMPLE		
Client Company Name	RAINBOW AND NATURE KOREA CO., LTD		
Client Address	205, Hanravivaldi Bldg, 15-10, Gangnam-daero 39-gil, Seocho-gu, Seoul, Korea		
Client Name	LEE BYONG KU	Client Tel / Fax	02-3473-2371
Lot No.		Date of Manufacture /Expiration Date	/
Test Purpose	For confirmation(in company)	Date of Issue	2021.12.22

Test Items and Results

1-Hexacosanol(mg/g)...............................5.51
1-Heptacosanol(mg/g)..............................5.81
1-Octacosanol(mg/g)...............................492.12
1-Tetracosanol(mg/g)..............................0.11
1-Nonacosanol(mg/g)...............................1.83
1-Triacontanol(mg/g)..............................12.63
1-dotriacontanol(mg/g)............................Not Detected
1-Tetratriacontanol(mg/g).........................Not Detected
Total Wax Alcohols(mg/g)..........................518.02
Lead(mg/kg).......................................Not Detected
Total Arsenic(mg/kg)..............................Not Detected
Cadmium(mg/kg)....................................Not Detected
Total Mercury(mg/kg)..............................Not Detected
Acetone(mg/kg)....................................Not Detected
Hexane(mg/kg).....................................0.0009
Coliforms...Negative

* The above test items and results complied with the test method notified by MFDS.
* Policosanol test items and results complied with the test method notified by Certificate of Functional Ingredient For Health/Functional Food No.2006-4

Note: 1. The above merchandise was submitted and identified by the client.
2. The results shown in this report refer only to sample tested and it does not cover the quality of all products.
3. No one can use this report for the purpose of test, advertisement and litigation without KAFRI's consent.
4. This report has no legal effect.
5. This report is not related to KS Q ISO/IEC 17025 and KOLAS Accreditation.

This certificate does not comply with the Act on Testing and inspection of Food and Drugs of Ministry of Food and Drug Safe.

Lee kyoung bok

Testing Personnel

Testing Manager

Korea Advanced Food Research Institute

Korea Advanced Food Research Institute

Data File: C:\Projects\Functional Food\Result\policosanol\2021\1208.rslt\025.dat
Method: C:\Projects\Functional Food\Method\Policosanol-1.met
Acquired: 12/9/2021 2:10:50 AM (GMT +09:00)
Sample Id: 11-22487
Operator: kafri169

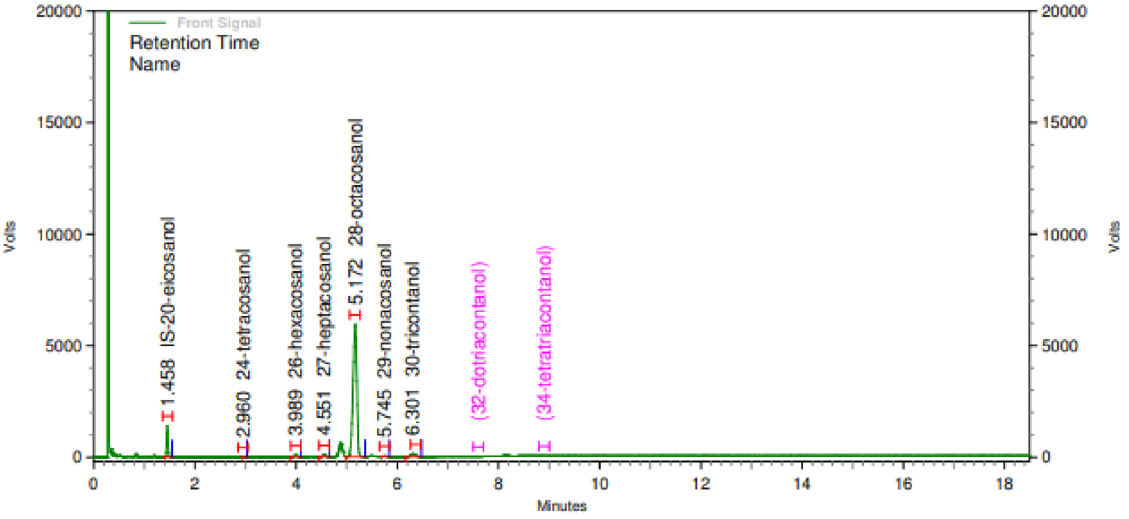

Front Signal Results

Name	Retention Time	Area
**IS-20-eicosanol**	1.458	22031541
**24-tetracosanol**	2.960	51915
**26-hexacosanol**	3.989	2498662
**27-heptacosanol**	4.551	2772372
**28-octacosanol**	5.172	236017055
**29-nonacosanol**	5.745	878106
**30-tricontanol**	6.301	5833943
**Totals**		270083594
